# Laparoscopic cytoreductive surgery and hyperthermic intraperitoneal chemotherapy for perforated low-grade appendiceal mucinous neoplasms

**DOI:** 10.1007/s00464-019-07349-x

**Published:** 2020-01-28

**Authors:** Haytham Abudeeb, Chelliah R. Selvasekar, Sarah T. O’Dwyer, Bipasha Chakrabarty, Lee Malcolmson, Andrew G. Renehan, Malcolm S. Wilson, Omer Aziz

**Affiliations:** 1 Colorectal and Peritoneal Oncology Centre, The Christie NHS Foundation Trust, Wilmslow Road, Manchester, M20 4BX UK; 2 Faculty Institute of Cancer Sciences, University of Manchester, Manchester, UK

**Keywords:** Laparoscopic, Cytoreductive surgery, Hyperthermic intraperitoneal chemotherapy, Low-grade appendiceal mucinous neoplasms

## Abstract

**Introduction:**

Cytoreductive surgery with hyperthermic intraperitoneal chemotherapy (CRS/HIPEC) is an established treatment for pseudomyxoma peritonei (PMP) from perforated low-grade appendiceal mucinous neoplasms (LAMN II). In a selected group of LAMN II patients without established PMP, CRS/HIPEC can be performed laparoscopically (L-CRS/HIPEC); however the short-term benefits and safety of this approach have yet to be determined. This study aims to determine the short-term outcomes from a series of L-CRS/HIPEC LAMN II patients compared to those who have undergone a similar open operation (O-CRS/HIPEC) for low-volume PMP.

**Methods:**

LAMN II patients undergoing L-CRS/HIPEC at a UK national peritoneal tumour centre were compared to O-CRS/HIPEC patients (peritoneal cancer index ≤ 7). Outcomes of interest included Clavien–Dindo complication grade, operative time, blood transfusions, high dependency unit (HDU) admission, length of hospital stay, and histopathological findings.

**Results:**

55 L-CRS/HIPEC were compared to 29 O-CRS/HIPEC patients (2003–2017). Groups were matched for age, sex, and procedures. Median operative time was 8.8 (IQR 8.1–9.5) h for L-CRS/HIPEC versus 7.3 (IQR 6.7–8) h for O-CRS/HIPEC (Mann–Whitney test *p* < 0.001). Post-operative HDU admission was 56% versus 97% (OR 0.04 95% CI 0.01–0.34) and median length of stay = 6 (IQR 5–8) versus 10 (IQR 8–11) days (*p* < 0.001) for L- versus O-CRS/HIPEC. Despite a normal pre-operative CT scan, 13/55 (23.6%) L-CRS/HIPEC patients had acellular mucin and 2/55 (3.5%) had mucin with epithelium present in their specimens. Residual appendix tumour was identified in 2/55 patients (3.6%). Clavien–Dindo Grade 1–4 complications were similar in both groups with no mortality.

**Conclusion:**

L-CRS/HIPEC for LAMN II takes longer; however patients have significantly reduced length of HDU and overall stay, without increased post-operative complications. A significant proportion of LAMN II patients undergoing L-CRS/HIPEC have extra-appendiceal acellular mucin with some cases demonstrating residual cellular epithelium from the LAMN II. The risk of these patients developing PMP without surgery is under current review.

Pseudomyxoma peritonei (PMP) is a rare condition with an annual incidence of 1.8 per 1 million population in the western world [1]. It most commonly arises from a low-grade appendiceal mucinous neoplasm (LAMN) [2] and presents with disseminated intraperitoneal mucinous tumour and free mucin. The established treatment for patients who present with PMP is open cytoreductive surgery and hyperthermic intraperitoneal chemotherapy (O-CRS/HIPEC), with a reported 20-year overall survival (OS) greater than 70% when a complete cytoreduction is achieved [3]. There are some patients who are diagnosed earlier with a LAMN at appendicectomy which is either confined to the appendix without evidence of perforation (LAMN I) or with appendiceal perforation and/or localised extra-appendiceal mucin (LAMN II) with or without neoplastic cells [4]. These patients have post-operative CT scans that do not show any clear evidence of extra-appendiceal disease and generalised PMP. There is evidence suggesting that such patients can go on to develop PMP through mucinous and epithelial dissemination into the peritoneal cavity; however the true potential is not clear, with this risk estimated to be somewhere between 5 and 40% [3, 5–9].

In PMP, the volume and distribution of disease is an important prognostic factor, with higher peritoneal cancer index (PCI) resulting in a significantly worse outcome. Patients with a PCI ≤ 10, for example, have a 10-year OS of 81% which is significantly higher than 55% for those with PCI = 21–30 [10]. This has led to a strategy to try and treat the disease as early as possible in its course, offering LAMN II patients at risk of developing PMP a laparoscopic CRS/HIPEC procedure (L-CRS/HIPEC) with risk-reducing intent [11].

A number of centres have now described their approach and outcomes from L-CRS/HIPEC to treat peritoneal surface malignancies of appendiceal [12, 13], ovarian [14, 15], colorectal [16], and mesothelial [17] origin. There remains however an absence of comparative data to quantify the benefit of the laparoscopic approach. This study aims to address this by presenting the short-term outcomes for a group of patients undergoing L-CRS/HIPEC for well-defined LAMN II lesions and comparing them to a group of patients undergoing O-CRS/HIPEC for low-volume PMP (PCI ≤ 7). It also aims to present the histological findings at L-CRS/HIPEC for this LAMN II patient group whose pre-operative CT scans showed no clear evidence of PMP.

## Materials and methods

### Patient population

A prospective database was used to collect information on patients who underwent CRS/HIPEC at a national peritoneal tumour centre in the United Kingdom between 2005 and 2017 and data analysed retrospectively [4, 18]. IRB approval and written patient consent were not needed for this study. Only patients undergoing L-CRS/HIPEC for LAMN II and O-CRS/HIPEC for PMP with PCI ≤ 7 were included. The Risk of Bias In Non-randomised Studies —of Interventions (ROBINS-I) assessment tool was used and classified as ‘moderate’ [19]. Patients referred from other hospitals after removal of their primary appendix tumour had their pathology specimens re-evaluated by pathologists at the national centre. Patients undergoing CRS/HIPEC for other peritoneal tumour types (adenocarcinoma, goblet cell carcinoids, ovarian tumours, primary peritoneal tumours, peritoneal mesothelioma, and peritoneal metastases from colorectal cancer) were excluded. All patients were discussed in a specialised peritoneal tumour multi-disciplinary team (MDT) meeting, where a consensus for management was reached based on review of CT scans, histology, prior operation details, and performance status. Patients undergoing L-CRS/HIPEC for LAMN II were only offered this approach if their CT scan did not show clear evidence of extra-appendiceal disease (PMP) and they had normal tumour markers (CEA, CA19-9, CA125).

### Operative technique

A standardised L-CRS/HIPEC closed technique was used and has been previously described [11]. The procedure in all cases included a greater and lesser omentectomy, excision of ligamentum teres, falciform ligament, cholecystectomy, umbilectomy, and relevant peritonectomies. In cases where the appendix stump was abnormal or present, this was removed with an excision of the caecal pole with a laparoscopic or open linear stapler. All women of fertile age wishing to have families were referred for egg harvesting and underwent fertility-preserving unilateral salpingo-oophorectomy. O-CRS/HIPEC using a semi-closed modified coliseum technique was used as has been previously described [20]. In all cases HIPEC was administered at a temperature of 42 °C for 90 min using Mitomycin C given in 3 equal boluses at a total dose of 35 mg/m^2^.

### Peri-operative care and follow-up

All O-CRS/HIPEC patients received full mechanical bowel preparation before surgery. In the peri-operative period they received an epidural, arterial line, central line, parenteral feeding, nasogastric tube, and spent the first post-operative night in our high dependency unit (HDU) having been extubated. L-CRS/HIPEC patients had a phosphate enema on the day of surgery, a nasogastric tube, and were allowed to drink fluids from the first day after surgery, gradually building up their oral intake. When the L-CRS/HIPEC technique was initially introduced, all patients were admitted to our HDU as a precaution; however after the first 25 cases, patients recovered on the ward after surgery. After discharge, patients were followed up every 6 months for 2 years and annually thereafter with CT abdomen/pelvis at 6, 12, 18, 24, 36, 48, 60, and 96 months accompanied by tumour markers (CEA, CA125, CA19-9).

### Data collection and outcome measures

All patients had their pathology reports, operation notes, and hospital records reviewed. Patient demographics and treatment history (prior surgery) were extracted. Operative data included date of procedure and PCI scores at CRS/HIPEC. Post-operative outcomes of interest included Clavien–Dindo complication grade, operative time, blood transfusion, HDU admission, and total length of hospital stay.

### Statistical analysis

Statistical analysis was undertaken using a* t* test for unpaired samples (operative time, HDU admission, and hospital stay). Chi-squared test was used for categorical values: hospital stay and short-term complications. The log-rank test was used to compare L- versus O-CRS/HIPEC and compensate for the time difference between both treatments. A standard linear model was used to predict the impact of the following variables on total operation time: number of procedures, sex, age and PCI Score. The difference in operation time for L- versus O-CRS/HIPEC was determined taking into account these variables.

## Results

### Patient groups

Between January 2003 and January 2018, 713 patients were referred to the national peritoneal tumour centre with a diagnosis of LAMN with or without established PMP. During this time 55 patients with LAMN II and no evidence of disease on a post-operative CT scan (at least 6 weeks after appendicectomy) were identified and chose risk-reducing L-CRS/HIPEC (as opposed to active surveillance with serial CT scans and tumour markers). In the same time period, 29 patients that had comparable O-CRS/HIPEC procedures for low-volume PMP (PCI ≤ 7) were identified. Table 1 outlines the baseline characteristics, operative, and short-term outcomes including Clavien–Dindo Grade 1–4 complications for the two groups. Patients were matched for age and sex.

**Table 1:** Patient demographics, operative outcomes, and post-operative complications

	Open CRS/HIPEC	Laparoscopic CRS/HIPEC	Odds ratio* (95% CI)	*P* value
No. of patients	29	55		
Male (%)	15 (52)	23 (42)		
Female (%)	14 (48)	32 (58)		0.386^†^
Median age (IQR) (years)	50 (43–62)	55 (44–64)		0.265^‡^
Median operation time (IQR) (h)	7.3 (6.7–8.0)	8.8 (8.1–9.5)		0.0001^†^
Median PCI score (IQR)	0 (0–1)	0 (0–2)		0.549^‡^
Blood transfusion (%)	1 (4)	1 (2)	0.52 (0.03–8.61)	0.641^†^
HDU admission (%)	28 (97)	30 (55)	0.04 (0.01–0.34)	< 0.001^†^
Median length of stay (IQR) ( days)	10 (8–11)	6 (5–8)		0.0001^‡^
C–D complications (90-day)				
Grade 1 (%)	0	1 (1.8)		
Grade 2 (%)	3 (10.3)	6 (10.9)		
Grade 3 (%)	2 (6.9)	1 (1.8)		
Grade 4 (%)	0	1 (1.8)		
Total (%)	5 (17.2)	9 (16.4)	0.93 (0.28–3.12)	1.000^†^

Any complications versus no complication

*IQR* interquartile range, *CI* confidence interval

^*^Unadjusted odds ratio expressed as L- versus O-CRS/HIPEC (as referent)

^†^Chi-squared or Fisher’s exact test as appropriate

^‡^Mann–Whitney test

The procedures performed as part of L- and O-CRS/HIPEC are shown in Table 2. The two groups were comparable with regard to their operative procedures. Not all patients underwent cholecystectomy as this had already been performed in some cases. Furthermore, women who underwent hysterectomy as part of L-CRS/HIPEC had this done for a separate pathology such as fibroids, endometrial, or cervical abnormalities. This was guided and recommended by a gynaecological specialist opinion.

**Table 2:** Operative procedures and post-operative histology for risk-reducing Open CRS/HIPEC and Laparoscopic CRS/HIPEC groups

Operative component	Open CRS/HIPEC *N* = 29 (%)	Laparoscopic CRS/HIPEC *N* = 55 (%)	Odds ratio* (95% CIs)	*P* value
Omentectomy	29 (100)	55 (100)	Not estimable	1.00
Cholecystectomy	25 (86)	53 (96)	4.24 (0.73–24.71)	0.175^†^
Excision of umbilicus	29 (100)	55 (100)	Not estimable	1.00
Excision of appendiceal stump	5 (17)	15 (27)	1.8 (0.58–5.58)	0.421^†^
Ileocaecectomy	0	2 (4)	Not estimable	0.542^†^
Women only (*n* = 46)				
Left salpingo-oophorectomy	8 (57)	23 (72)	1.92 (0.52–7.10)	0.327^†^
Right salpingo-oophorectomy	10 (71)	25 (78)	1.43 (0.43–5.97)	0.713^†^
Bilateral salpingo-oophorectomy	2 (14)	8 (25)	2.00 (0.37–10.91)	0.699^†^
Hysterectomy	5 (36)	4 (13)	0.26 (0.06–1.17)	0.106^†^

*CI* confidence interval

^*^Unadjusted odds ratio expressed as laparoscopic versus open (as referent) CRS/HIPEC

^†^Chi-squared or Fisher’s exact test as appropriate

**Fig. 1:**
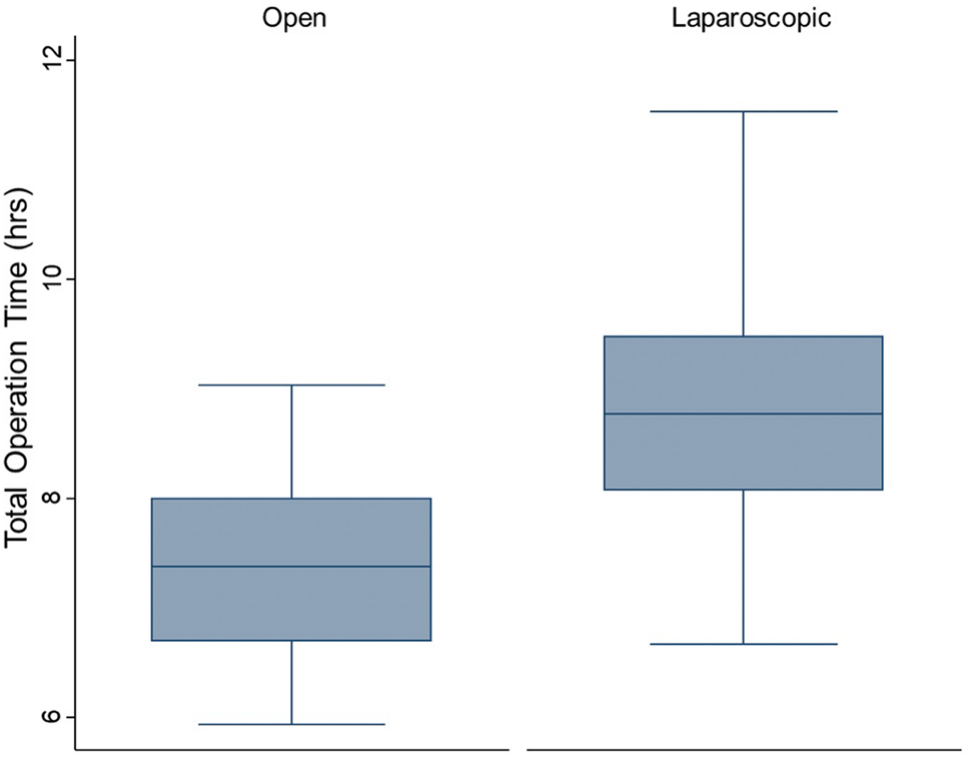
Box plot comparing operative time for L-CRS/HIPEC to O-CRS/HIPEC

**Fig. 2:**
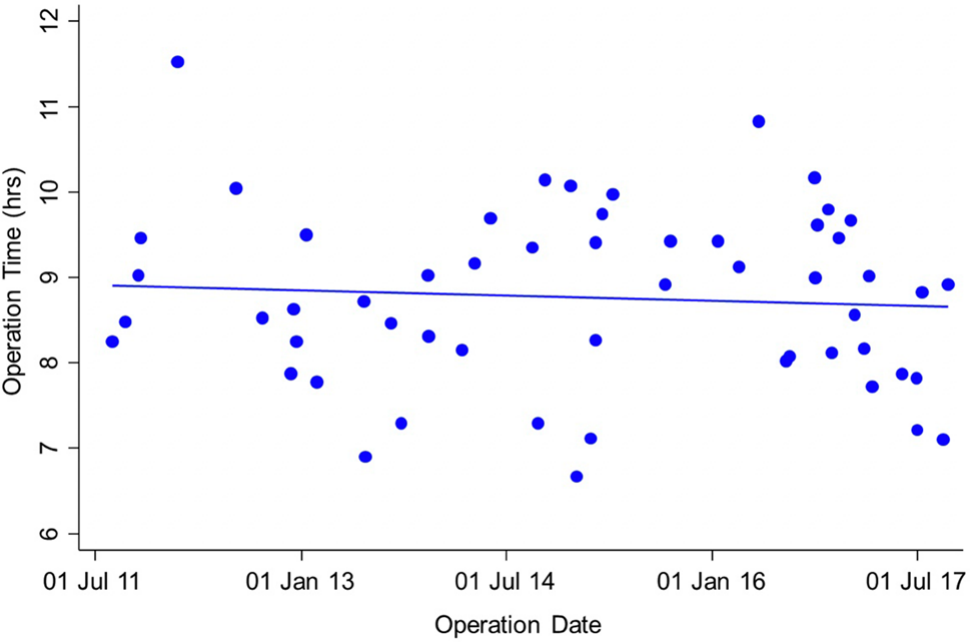
Graph showing operative time for L-CRS/HIPEC over time

**Fig. 3:**
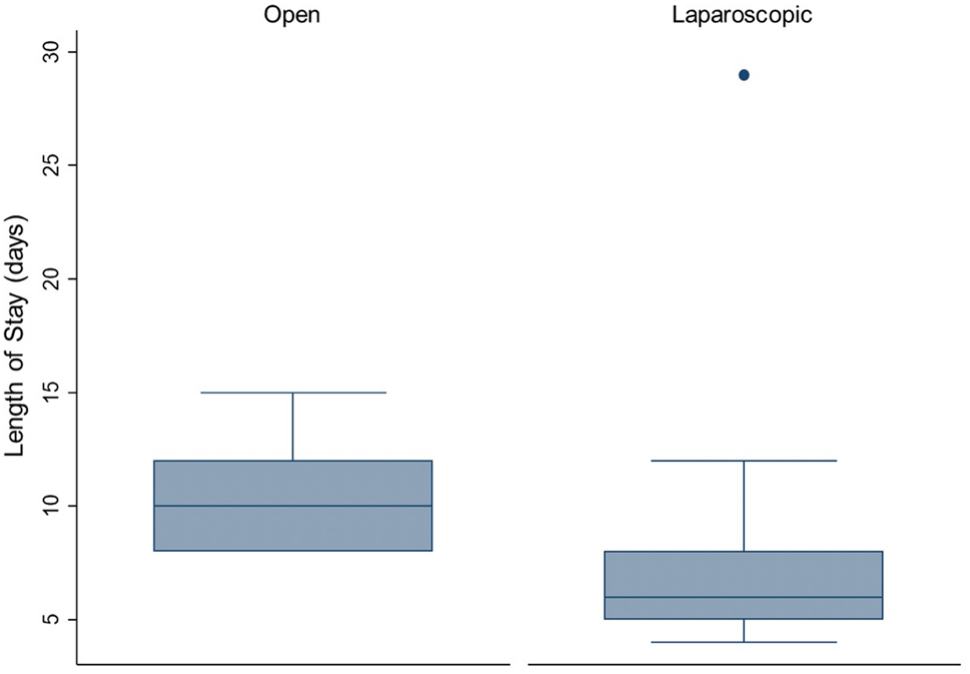
Box plots comparing median post-operative length of stay for L-CRS/HIPEC versus O-CRS/HIPEC

Patients undergoing L-CRS/HIPEC had a significantly longer median operative time of 8.8 (IQR 8.1–9.5) h, versus 7.3 (IQR 6.7–8.0) h for O-CRS/HIPEC (Mann–Whitney test* p* < 0.001), as shown in Fig. 1. Among the 55 cases over the study period however, the mean operative time reduced from 8 h and 55 min to 8 h and 39 min (regression* p* = 0.608) as demonstrated in Fig. 2. There was no significant difference in post-operative complications (17.2% with O-CRS/HIPEC, 16.4% with L-CRS/HIPEC). The median post-operative length of stay was significantly higher at 10 days (IQR 8–11) for O-CRS/HIPEC with versus 6 days (IQR 5–8) with L-CRS/HIPEC (Mann–Whitney test* p* > 0.001) as shown in Fig. 3.

Review of operative notes and histology specimens for the L-CRS/HIPEC patients demonstrated that despite a pre-operative CT scan showing no evidence of residual disease, acellular mucin was identified in 13/55 patients (23.6%) and mucin with epithelium was identified in 2/55 (3.6%). A residual appendix stump tumour was identified in 2/55 patients (3.6%).

## Discussion

This study has demonstrated that L-CRS/HIPEC can be performed safely with acceptable short-term outcomes. Despite the increased operative time, Grade 1–4 complication rates and the need for blood transfusions were similar in both groups and the benefits of the laparoscopic approach include a shorter length of stay without the need for the patient to be admitted to a high dependency unit in most cases. Just like other forms of laparoscopic surgery, there is a significant learning curve associated with L-CRS/HIPEC as demonstrated by the reduction in operative time over the study period. Components such as the omentectomy in particular are technically challenging and we have adopted a mentorship approach to these cases for new members of the team taking on the procedure. Clearly the group of patients undergoing L-CRS/HIPEC in this study had very low volume or no visible disease, and if L-CRS/HIPEC is to be used for more advanced cases, there needs to be very careful case selection which may improve with newer imaging techniques such as magnetic resonance imaging. Furthermore a database of these cases should be maintained to allow review of long-term outcomes.

The finding that 32.5% of patients undergoing L-CRS/HIPEC for LAMN II were found to have peritoneal acellular mucin and 3.6% of patients had peritoneal mucin with cells was unexpected. Furthermore 3.6% of patients had residual tumour at the appendicectomy stump. This is an important finding when considering the quantification of risk for patients with incidental LAMN at appendicectomy going on to develop PMP. There are two distinct groups of patients in which this risk has to be considered. The first is patients with LAMN confined to the appendix with no evidence of leakage of mucin outside the appendix (LAMN I) [3, 6, 7]. The limited literature available suggests that these patients have a very low risk of developing PMP, and our practice has been to offer these patients surveillance though serial CT scans and tumour markers (CEA, CEA125, CA19-9). The second group are patients with a LAMN that has ruptured with evidence of leakage of acellular mucin onto the serosal surface (LAMN II) diagnosed at appendicectomy. The limited literature has suggested these patients do have a 4–8% risk of developing PMP, which is higher in 33–75% for those with cellular material in the mucin or on the surrounding peritoneum [6, 7].

Most patients with LAMN II are diagnosed following an appendicectomy performed by surgeons with little or no experience of peritoneal neoplasia. Hence it is unlikely that the peritoneal cavity has been sufficiently evaluated at the initial appendicectomy whether performed laparoscopically or open. It is therefore our practice to request a post-operative CT scan of the abdomen and pelvis with oral and intravenous contrast at least 6 weeks after the appendicectomy to allow for post-operative changes to resolve and to look for residual or disseminated disease. This study has demonstrated that 36.1% of LAMN II patients with a ‘normal’ post-appendicectomy CT scan discussed in a specialised peritoneal tumour MDT setting have evidence of disease at laparoscopy. Although 23.6% of these patients had acellular mucin only, the risk of such patients going on to develop PMP with or without the procedure needs to be quantified.

While offering L-CRS/HIPEC for LAMN II, it is important to consider a number of factors: First, the procedure is associated with morbidity, as it is a major operation with a 3.6% risk of Grade 3–4 complications. Second, because it may take time (5–10 years) for PMP to develop, the age at diagnosis is important to consider. The median age was 55 years in our L-CRS/HIPEC group. Finally, issues of fertility need to be taken into account. We have addressed this through offering pre-menopausal women the option of egg harvesting and fertility preservation through unilateral oophorectomy with avoidance of hysterectomy. This strategy has seen in patients who have undergone L-CRS/HIPEC go on to have children.

There are a number of limitations to this study. First, is the small sample size which is inevitably a challenge when studying rare appendiceal neoplasms. Addressing this will only be possible through multi-centre collaboration. Second, is the absence of long-term follow-up (our median follow-up for this L-CRS/HIPEC is 27.6 months which is short to draw any conclusions). As time goes on our practice will allow the prospective evaluation of this group into the future and help generate debate about the place for laparoscopic techniques in low-risk PMP patients. Finally, our study has demonstrated that CT scanning has limitations when looking for low-volume peritoneal disease making early recurrence difficult to diagnose, suggesting the need to look at MRI as an alternative imaging modality in these patients.

This study has demonstrated that L-CRS/HIPEC is a strategy that can be used safely in patients with LAMN II with short-term outcomes that are acceptable compared to O-CRS/HIPEC. Further collaborative multi-centre research on early LAMNs is needed to further define the risks of these patients going on to develop PMP. Education of clinicians about LAMN and PMP will lead to its earlier detection and referral to experienced peritoneal tumour centres. This is desirable particularly when it is considered that CRS/HIPEC for higher-volume PMP is associated with significantly poorer short- and long-term outcomes.
